# Poor risk–benefit ratio of gastrointestinal endoscopy for screening prior to heart or lung transplantation

**DOI:** 10.1007/s00464-025-11678-5

**Published:** 2025-03-31

**Authors:** Hanno Ehlken, Jana Foerster, Markus Johannes Barten, Malte Issleib, Thomas Rösch, Hermann Reichenspurner, Guido Schachschal

**Affiliations:** 1 Department of Interdisciplinary Endoscopy, University Hospital Hamburg-Eppendorf, Martinistrasse 52, 20246 Hamburg, Germany; 2 Department of Cardiovascular Surgery, University Medical Center Hamburg-Eppendorf University Heart & Vascular Center, Hamburg, Germany; 3 Department of Anesthesiology, University Hospital Hamburg-Eppendorf, Hamburg, Germany

**Keywords:** Heart transplantation, Lung transplantation, Tumor screening, Gastrointestinal endoscopy

## Abstract

**Background:**

Routine pre-transplant evaluations for heart or lung recipients often include upper and lower gastrointestinal endoscopies. However, given that these patients frequently have significant and multiple comorbidities, the risk–benefit ratio of endoscopy with sedation remains uncertain.

**Methods:**

We conducted a retrospective chart review of all patients who underwent esophagogastroduodenoscopy (EGD) and/or colonoscopy as part of the pre-transplant evaluation for heart (HTX) or lung transplantation (LuTX) at our center over a 10-year period. All procedures were performed with either anesthesiologist-assisted sedation or general anesthesia (72%) or physician-assisted sedation (28%). The primary outcomes included the prevalence of pre/neoplastic lesions and the rate of adverse events, classified according to the American Society of Gastrointestinal Endoscopy.

**Results:**

A total of 315 patients (70% male, median age 55 years) were included, with ASA grades 3 (31%) and 4 (69%). Of these, 90% underwent both EGD and colonoscopy. While no neoplasia or findings relevant to transplantation were detected on EGD, a single rectal malignant pedunculated polyp was identified and curatively removed via colonoscopy (G2 pT1 L0 V0 R0). The adenoma detection rate during colonoscopy was 25%, including a 7% rate of advanced adenomas, with six additional lesions (< 10 mm) exhibiting high-grade dysplasia. Conversely, severe adverse events occurred in 2.5% of cases, involving both cardiovascular and colonoscopy-related complications. These included two cases of severe hypotension and bradyarrhythmia requiring cardiovascular resuscitation, with one resulting in death (0.3% mortality). Additionally, one splenic rupture, two colonic perforations, and two cases of severe post-polypectomy bleeding were observed.

**Conclusion:**

The routine requirement for upper and lower gastrointestinal endoscopy as a screening measure before heart or lung transplantation should be reconsidered due to its unfavorable risk–benefit profile. An individualized approach should be taken.

**Supplementary Information:**

The online version contains supplementary material available at 10.1007/s00464-025-11678-5.

Post-transplant malignancy is a well-recognized major cause of morbidity and mortality in heart (HTX) and lung transplant (LuTX) recipients [1–3]. Since advanced malignancy is generally an absolute contraindication for transplantation, thorough pre-transplant screening is essential. Like other organ transplant candidates, patients awaiting heart or lung transplantation undergo a series of medical evaluations to rule out contraindications. Among these, upper and lower gastrointestinal endoscopies are commonly performed, though their indications and age cut-offs vary between countries and centers.

Screening and diagnostic gastrointestinal (GI) endoscopy, including colonoscopy and, in some countries, esophagogastroduodenoscopy (EGD), is generally considered a safe procedure in otherwise healthy individuals [4, 5]. However, the risk–benefit profile may differ significantly in patients with advanced heart and lung disease.

To assess the risk–benefit profile of routine screening upper and lower GI endoscopy as part of our center’s pre-transplant checklist, we analyzed a large patient cohort over a 10-year period. In accordance with our department’s protocol for high-risk patients, sedation was administered by an anesthesiology support team in most cases.

## Patients and methods

Using documentation systems and chart reviews from the endoscopy, cardiology, and transplantation departments, we included all adult patients referred to the Department for Interdisciplinary Endoscopy at University Hospital Hamburg-Eppendorf for EGD or colonoscopy as part of their heart and/or lung transplant evaluation over a 10-year period. The study was conducted in accordance with the national and local ethics guidelines and was reviewed by the local medical ethics committee (Ärztekammer Hamburg, 2021–300130-WF). Individual patient consent was waived due to the study’s observational and retrospective nature.

Patient and endoscopic characteristics were assessed through a review of digital charts. Collected data included patient age, underlying heart and/or lung disease, ASA classification, details of endoscopic procedures (including duration, interventions, findings, and complications), histologic results, post-procedural course, and follow-up information. A six-month follow-up was conducted for all patients to gather information on their general health and transplantation status.

### Statistics

This retrospective study was primarily exploratory. Data were obtained from digital patient charts (Soarian Clinicals, Cerner Health Care, Berlin, Germany). Statistical analyses were performed using GraphPad Prism v6.0e (GraphPad Software, San Diego, USA), while data management and analysis were conducted using MS Excel 2008 for Mac (Microsoft Corp., Redmond, WA, USA).

### Definitions

#### Key findings from esophagogastroduodenoscopy (EGD)

Stage of reflux disease, hiatal hernia, candidiasis, polyps, varices, Barrett metaplasia including Prag classification, hypertensive gastropathy, erosive lesions ≥ 3 mm, ulcers, neoplastic lesions, submucosal lesions, and inflammatory processes.

#### Key findings from colonoscopy

Neoplastic findings included cancers, precancerous lesions (adenomas), and other tumors, such as neuroendocrine tumors. The adenoma detection rate (ADR) was defined as the proportion of patients with at least one colorectal adenoma, including sessile serrated adenomas (SSA). Advanced adenomas were classified as those with high-grade dysplasia, a size > 1 cm, and/or a villous component.

### Therapeutic interventions

In accordance with standard practice, polyps larger than 3 mm were removed using hot polypectomy, with or without prior submucosal saline injection. Smaller polyps were typically resected using standard forceps. Resected polyps were fixed in paraformaldehyde solution and sent to the pathology department for analysis. Mucosal biopsies were also collected using standard forceps.

### Adverse events

To assess incidents and adverse events, we classified their severity based on recommendations from an American Society of Gastrointestinal Endoscopy (ASGE) workshop. Adverse events were categorized as mild, moderate, severe, or fatal [6]. Minor incidents without significant consequences were not classified as adverse events [6].

## Results

Over a ten-year period, 315 adult candidates for heart and/or lung transplantation underwent EGD and colonoscopy (median age: 55 years, range: 20–76; 70% male). Basic patient characteristics and details of the endoscopic examinations are summarized in Table 1. Notably, most patients were classified as ASA 4. All underwent EGD and colonoscopy as part of routine screening, with only a few reporting mild reflux symptoms. Approximately, 90% of patients underwent both procedures, with 88% having them performed sequentially on the same day.

**Table 1:** Patient characteristics

Total number reviewed, *n*	315
Male, *n*	221 (70%)
Age at Scan, median years (range)	55 (20–76)
Patients < 55 years of age, *n*	152 (48%)
Patients < 50 years of age, *n*	88 (28%)
HTX candidate, *n*	223 (70.8%)
LUTX candidate, *n*	90 (28.6%)
HTX plus LUTX candidate, *n*	2 (0.6%)
ASA 3	97 (30.8%)
ASA 4	218 (69.2%)
Anesthesia by anesthetist	227 (72%)
Anesthesia by physician / 2nd endoscopist	87 (28%)
Duration of endoscopy, minutes mean (range)	34 (15–155)
Index endoscopies ^§^	314
EGD plus colonoscopy	282 (89.8%)
EGD plus colonoscopy same day	277 (88.2%)
EGD plus colonoscopy different days	5 (1.6%)
EGD only	25 (8%)
Colonoscopy only	7 (2.2%)
Endoscopies total ^§§^	333

^§^One examination was not performed because of complication with colonoscopy preparation

^§§^Total numbers including repeated examinations due to complication or poor preparation

The mean interval between endoscopy and transplantation was 296 days (range: 4–1578 days, median: 183 days). This duration varied between heart transplant candidates (*n* = 90; mean: 274 days, range: 4–1578) and lung transplant candidates (*n* = 30; mean: 360 days, range: 6–1492). During the follow-up period, 38.1% of patients (120 of 315) ultimately underwent transplantation.

### EGD results

Results are summarized in Table 2. No EGD findings led to an absolute contraindication for transplantation. Barrett’s metaplasia was detected in 28 patients (9.1% of EGDs), none of whom had dysplasia on biopsy. Most cases (86%) were classified as short-segment Barrett’s esophagus (< 3 cm). *H. pylori*-positive gastritis was identified in six patients (2%), and no ulcerations were observed (0 of 307 patients). Additionally, two patients (0.7%) had small (≤ 3 mm) low-grade duodenal adenomas. Further details are provided in Supplementary Table S1, although they were not considered clinically significant. No therapeutic interventions were required.

**Table 2:** Age-dependent ADR in colonoscopy

Total index colonoscopies, *n*	289 (100%)
Age ≥ 55 years	157 (54.3%)
Age < 55 years	132 (45.7%)
Age < 50 years	68 (23.5%)
ADR, entire cohort	25.3%
ADR, ≥ 55 years	34.4%
ADR, < 55 years	14.4%
ADR, < 50 years	8.8%
Carcinoma,* n*	1 (0.3%)
Carcinoma ≥ 55 years, *n*	1 (0.6%)
HGIN,* n*	7 (2.4%)
HGIN ≥ 55 years, *n*	5 (3.2%)
HGIN < 55 years, *n*	2 (1.5%)
HGIN < 50 years, *n*	1 (1.5%)
*LGIN, n*	57 (19.7%)
LGIN ≥ 55 years, *n*	42 (26.8%)
LGIN < 55 years, *n*	15 (11.4%)
LGIN < 50 years, *n*	3 (4.4%)
SSA,* n*	8 (2.8%)
SSA ≥ 55 years, *n*	6 (3.8%)
SSA < 55 years, *n*	2 (1.5%)
SSA < 50 years, *n*	2 (2.9%)

### Colonoscopy results

A total of 289 transplant candidates (92%) underwent colonoscopy, with most (277 of 289, 96%) having the procedure immediately after EGD (see Table 1). The cecal intubation rate was 95.8%. Bowel preparation was generally suboptimal in this cohort, leading to early termination of six colonoscopies due to inadequate visualization. Three of these patients underwent a repeat examination within 7–15 days. As with EGD, no findings during colonoscopy resulted in an absolute contraindication for transplantation. Findings are summarized in Table 2 and detailed in supplementary Table S2.

Colonoscopy revealed no absolute contraindications for transplantation. Diverticulosis was identified in 67 patients (23.9% of the total colonoscopy cohort), with a prevalence of 21% (43 patients) among heart transplant (HTx) candidates and 28.6% (24 patients) among lung transplant (LuTx) candidates. One patient exhibited mild endoscopic signs of diverticulitis but remained clinically asymptomatic. Further details are provided in supplementary Table S2.

Adenoma detection rate (ADR) and rate of advanced adenomas (aADR): The adenoma detection rate (ADR) was age dependent (Table 2). Despite suboptimal bowel preparation, an ADR of 34.4% was achieved in patients aged ≥ 55 years. In patients younger than 55 and 50 years, ADRs were 14.4% and 8.8%, respectively. Adenomas with focal high-grade intraepithelial neoplasia (HGIN) were identified in seven patients (2.4%), while sessile serrated adenomas (SSA) were found in eight patients (2.8%). Advanced adenomas were detected in 10% (16 of 157) of patients aged ≥ 55 years (Supplementary Table S4). Among patients younger than 55 and 50 years, the advanced adenoma detection rate (aADR) was 3% (4 of 132) and 1.5% (1 of 68), respectively (Supplementary Table S3).

One 57-year-old patient (0.3%) had a 14-mm pedunculated rectal polyp, which was removed via conventional hot snare polypectomy and diagnosed as early-stage cancer (G2 pT1, L0, V0, R0). Notably, the same patient was also diagnosed with early-stage bladder cancer (pTa G3) during screening cystoscopy as part of the transplant evaluation. Among patients with neoplastic polyps (including cancer, HGIN, SSA, or LGIN), 43.8% had ≥ 2 lesions. Nearly three-quarters of all polyps were detected in patients aged ≥ 55 years. Detailed findings on removed polyps are summarized in Supplementary Table S4.

### Complications of EGD and colonoscopy

A total of 32 patients (10.2%) experienced relevant adverse events (AEs) related to endoscopy (Table 3). Nearly half (*n* = 15, 46.9%) of the AEs were classified as mild, while 28.1% (*n* = 9) were moderate and 21.9% (*n* = 7) were severe. There was one fatal event (0.3%). All severe AEs were associated with either sedation or colonoscopy and occurred within the anesthetist-assisted group (Table 4). One patient died after unsuccessful resuscitation for electromechanical dissociation during the procedure. Another patient was deemed ineligible for lung transplantation after prolonged intensive care treatment and multiple surgeries due to cecal perforation following polypectomy. She was eventually discharged to a weaning hospital and later received home nursing care.

**Table 3:** Adverse events in transplant candidates

All Patients, *n*	315 (100%)
Adverse Events (all)	32 (10.2%)
Mild	15 (4.8%)
Moderate	9 (2.9%)
Severe	7 (2.2%)
Fatal	1 (0.3%)
Anesthesia-associated Adverse Events§ (all)	7 (2.2%)
Mild	4 (1.3%)
Moderate	0
Severe	2 (0.6%)
Fatal	1 (0.3%)
Adverse Events during EGD (all)	0
Mild	0
Moderate	0
Severe	0
Fatal	0
Adverse Events during colonoscopy (all)	4 (1.4%)
Mild	2 (0.7%)
Moderate	0
Severe	2 (0.7%)
Fatal	0
Adverse Events after EGD, Colonoscopy (all)	16 (5.1%)
Mild	6 (1.9%)
Moderate	7 (2.2%)
Severe	3 (1.0%)
Fatal	0
Adverse Events before Endoscopy (all)	5 (1.6%)
Mild	3 (1%)
Moderate	2 (0.6%)
Severe	0
Fatal	0

^§^Total *n* = 311 (3 patients without sedation)

**Table 4:** Serious adverse events of endoscopies in patients evaluated for HTX/LUTX

Patient No	Age (years)	Sex	Evaluation for	Adverse event	Follow-up
1	51	m	HTX; ICM	Severe hypotension during anesthesia induction (MAP < 30), no response, epinephrine injection, ROSC, transfer to ICU	Recovery; later endoscopy
2	58	f	LUTX; LF, PHT	Electromechanic dissociation during colonoscopy. Prolonged resuscitation, transfer to ICU, and ECMO implantation. Death 6 days after endoscopy due to SIRS and heart failure	Death
3	59	m	HTX; DCM	Loss of spontaneous circulation upon anesthesia induction. Mechanical resuscitation (30 s), epinephrine, ROSC	Recovery; later endoscopy
4	54	f	LUTX; COPD	Cecum perforation, emergency cecal resection, peritonitis, sepsis, 3 re-laparotomies, and vacuum therapy for secondary wound healing, weaning in hospital, and home nursing	Evaluated as not transplantable
5	56	m	HTX; DCM	Splenic rupture during colonoscopy. Emergency splenectomy	Recovery; HTX later
6	60	m	HTX; ICM	Rectal polypectomy. Prolonged bleeding over 12 days. Seven RBC transfusions. Transfer to IMC. 4 re-colonoscopies with final fibrin sealant therapy due to intramural hematoma	Recovery; later endoscopy
7	52	m	HTX; ICM	Cecal, sigmoid, and rectal polypectomy, diverticulosis. Left sided local peritonism after colonoscopy with ever and chills. Leukocytosis, 28 × 10*9/L. CT without evidence of perforation. Antibiotic + volume treatment. IMC	Recovery
8	52	m	HTX; DCM	Rectal and sigmoid polypectomy. Hemorrhagic shock. RBC transfusion and emergency colonoscopy with clipping	Recovery

Additionally, one mild anesthesia-related event occurred in a patient under physician- or endoscopist-assisted sedation, where transient oxygen desaturation required short-term mask ventilation. Further details on severe complications are provided in Table 3.

## Discussion

Endoscopic procedures in generally healthy individuals have very low rates of significant complications [4, 6, 7]. However, the indication for endoscopy must be carefully considered, especially in high-risk patients, such as those undergoing evaluation for heart (HTX) or lung transplantation (LuTX). At our hospital, as well as other German centers, screening EGD and colonoscopy are standard assessments for all adult HTX and LuTX candidates. However, limited data exist on the risks and benefits of these procedures in this specific patient group. A recent retrospective study of 213 transplant candidates (heart: *n* = 53, lung: *n* = 160) reported a favorable risk profile for upper and lower endoscopy in these patients. However, only 61% and 35% underwent upper and lower endoscopy, respectively, before transplantation, and the study did not differentiate between screening endoscopy for transplantation and endoscopy performed for symptoms or complications [8]. Another paper on screening colonoscopy in 808 patients before liver transplantation found an increased rate of renal failure (3.8%) and gastrointestinal bleeding (2.9%) after the procedure as compared to controls. Advanced neoplasia and cancer were found in the pre-transplant group in 5.6% and 0.4% of cases, respectively [9].

To address the knowledge gap on the value of routine endoscopy before HTX and LuTX, we retrospectively evaluated the risk–benefit ratio of screening EGD and colonoscopy in HTX and LuTX candidates. The vast majority of procedures were performed under anesthesiologist-administered sedation, ensuring the highest safety standards for these patients, all of whom were classified as ASA III or IV [10–13].

Our study, conducted in a large cohort of pre-transplant candidates, nearly all of whom underwent both upper and lower GI endoscopy, found that the risk–benefit profile of routine GI endoscopy was not favorable. The findings do not support the *universal* use of these procedures before transplantation in all high-risk patients. Instead, an individualized approach should be considered, incorporating informed consent and shared decision-making. 

Endoscopic findings were largely as expected and could have been detected through age-appropriate screening after transplantation. However, the risk of severe adverse events was relatively high, far exceeding that of routine endoscopy patients, at least within this cohort.

As a retrospective single-center study, our analysis has inherent limitations, including potential variations in risks and findings across other centers. While our cohort (*n* = 315) is relatively large for this specialized patient group, it remains insufficient for accurate risk calculations of rare events, such as cancers (one rectal, none in the upper GI tract), which could temporarily preclude transplantation.

Individual risk assessment—considering symptoms, family history, clinical findings, and results from less invasive tests—should guide the decision on whether a patient requires endoscopy before transplantation. Notably, as expected, only 40% of patients in our cohort ultimately underwent transplantation. The necessity of routine pre-transplant endoscopy should be further discussed with transplant surgeons, and patients should be actively involved in a shared decision-making process. This should also include consideration of alternative screening methods, such as fecal immunochemical testing (FIT) instead of colonoscopy, despite inherent limitations.

The risk of malignancy increases after transplantation due to various factors, including immunosuppression. As outlined by Chapman et al. [14], “post-transplant malignancies account for 10%–47% of deaths in solid organ recipients, and mortality from these malignancies progressively increases with postgraft survival. According to U.S. Organ Procurement and Transplantation Network (OPTN) and United Network for Organ Sharing (UNOS) data, post-transplant malignancy-related mortality at 5–10 years is 14.5% for kidney, 18.7% for liver, and 21.5% for heart transplant recipients” [14]. While overall cancer risk in transplant recipients is two- to threefold higher than in the general population, certain virally driven malignancies carry an even greater risk. Colorectal cancer (CRC) shows a reported 1.2–3 fold increase, as confirmed by more recent analyses from the U.S.A. and China. However, findings remain variable, with some smaller studies reporting no increased CRC risk but a higher prevalence of adenomas.

These findings strongly support the need for screening colonoscopy after transplantation rather than as a routine pre-transplant procedure.

Regarding endoscopic findings, no clinically relevant lesions were detected on upper GI endoscopy, and colonoscopy findings aligned with standard risk-adapted expectations. Consequently, we could not identify a subgroup of high-risk patients who would specifically require pre-transplant endoscopy. This suggests that, at least for colorectal adenomas, transplant candidates have a standard risk profile for upper and lower GI lesions. However, the sample size was too small to assess colorectal cancer (CRC) risk accurately. Notably, in most Western countries, upper GI endoscopy is not part of routine cancer screening.

A recent review of clinical practice guidelines for cancer evaluation in solid organ transplant candidates [15] identified three guidelines addressing heart and/or lung transplantation. While all recommended screening for colorectal cancer—alongside cervical, breast, and prostate cancer—none included upper GI endoscopy. Furthermore, these guidelines did not grade the available evidence supporting their recommendations and their suggested screening tests, including colonoscopy, largely following the general population screening criteria for each country.

Post-transplant studies have reported an increased risk of CRC, likely due to immunosuppression and/or chronic viral infections. Although CRC risk is moderately elevated (2–5 fold) after transplantation, it is not among the leading post-transplant malignancies [16]. Therefore, screening could reasonably be initiated after transplantation when the patient's cardiovascular risk profile is expected to improve. In patients who do not undergo transplantation—65% within a median of 28 months in our cohort—prognosis may be poor, making routine cancer screening less relevant.

In our cohort, severe adverse events occurred in 2.2% of cases due to endoscopic procedures, while no absolute contraindications for transplantation were identified. An additional 2.9% of patients experienced complications categorized as moderate based on ASGE guidelines [6]. However, adverse events considered moderately severe in otherwise healthy individuals may pose greater concerns in this critically ill population, where 69% were classified as ASA IV. These findings suggest that routine screening EGD and colonoscopy should not be universally performed in HTX and LuTX candidates. The observed rate of significant adverse events (> 5%) underscores the need for individualized risk stratification.

For the general population, screening colonoscopy is recommended for individuals aged 50–55 years. In our cohort, patients younger than 55 had lower ADRs than older individuals, while EGD failed to provide any clear benefit. An alternative approach could involve identifying high-risk individuals through a thorough assessment of personal and family history. Patients with a normal colonoscopy within the past seven years should not undergo repeat screening. Fecal blood tests may have limited utility in this cohort due to the high prevalence of anticoagulant or antiplatelet use, but more data are required.

Notably, severe or fatal adverse events in our study were exclusively linked to sedation/anesthesia complications or colonoscopy. Screening sigmoidoscopy in awake patients, rather than full colonoscopy under sedation, could reduce risks while still providing valuable information. Additionally, smaller polyps without high-risk endoscopic features could be left untreated and resected more safely post-transplant.

Our findings also raise legal concerns for endoscopists. In many cases, the indication for endoscopy in transplant candidates may be driven primarily by administrative protocols rather than clear clinical necessity. As a result, endoscopists may be obligated to perform procedures without a compelling medical indication. Given the significant rate of serious adverse events, the legal framework surrounding these procedures must be clearly defined. Informed consent presents an additional challenge, as endoscopists may be required to perform routine procedures despite an unfavorable risk–benefit profile. If the evidence does not strongly support pre-transplant endoscopy, obtaining truly informed consent becomes complex.

Ultimately, as in other fields of medicine where higher-risk procedures are performed (e.g., ERCP), professional societies and guidelines must be developed to support clinical decision-making. This should involve all relevant specialties involved in transplantation, as well as patient advocacy groups, to ensure a balanced and evidence-based approach.

## Supplementary Information

Below is the link to the electronic supplementary material.

Supplementary file1 (PDF 58 KB)
