# Supplementary material for: Poor risk–benefit ratio of gastrointestinal endoscopy for screening prior to heart or lung transplantation
Source: Surg Endosc. 2025 Mar 31;39(5):3106–12. doi: 10.1007/s00464-025-11678-5 (PMC12041142; doi:10.1007/s00464-025-11678-5)
Supplement: Supplementary file 1 — Supplementary file1 (PDF 58 KB) [file 464_2025_11678_MOESM1_ESM.pdf]

Suppl. Table 1. Detailed findings in EGD

Esophagus	
Esophageal reflux disease	41 (13%)
LA grade A	40 (98%)
LA grade B	1 (2%)
Hiatal hernia	42 (13.7%)
Small (≤3cm)	33 (79%)
Large (>3cm)	9 (21%)
Candida	14 (4.6%)
Papilloma	1 (0.3%)
Varices, low grade	2 (0.7%)
Varices, high grade	0
Barrett metaplasia	28 (9.1%)
Short segment Barrett	24 (86%)
Long segment Barrett	4 (14%)
Dysplastic Barrett	0
Stomach	
Hypertensive gastropathy	6 (2%)
Gastric Ulcer	3 (1%)
Erosive lesion ≥3mm	7 (2.3%)
H. pylori positive stomach biopsy §	6 (2%)
Duodenum	
Duodenitis	15 (4.9%)
Duodenal Adenoma	2 (0.7%)
Adenoma ≤3mm	2 (100%)
Adenoma >3mm	0
Duodenal diverticula	2 (0.7%)
Duodenal non dysplastic polypoid lesion	3 (1%)
Duodenal submucosal lesion	2 (0.7%)

§ in 116 cases (37%) aspects of gastritis were seen endoscopically

Suppl. Table 2. Detailed findings in Colonoscopy

Cecum intubation	(95.8%)
Ileum intubation	108 (37.4%)
Ulcer Ileocecal valve	1 (0.7%)
Normal Ileum	107 (99%)
Diverticulosis	
All	67 (23.2%)
HTx candidates	43 (21%)
Age, median (range)	55 (42-74)
LuTx candidates	24 (28.6%)
Age, median (range)	55 (37-65)
Diverticulitis	1 (0.3%)
Unspecific colitis (histology)	3 (1%)
Proctitis	1 (0.3%)
Angiodysplasia	3 (1%)
Rectal varices	1 (0.3%)
Hemorrhoids	24 (8.3%)
Grade I + II	20 (7%)
Grade III +IV	4 (1.3%)

Suppl. Table 3. Detection of advanced adenomas in colonoscopy

	**All n=289**	**≥ 55 years n=157**	**< 55 years n=132**	**< 50 years n=68**
**Advanced Adenoma**				
>1cm	5	4	1	0
LGIN tubulovillous	7	7	0	0
LGIN > 1cm + villous	1	0	1	0
HGIN	6	4	2	1
>1cm + HGIN	1	1	0	0
Total	20 (6.9%)	16 (10.2%)	4 (3%)	1 (1.5%)

Suppl. Table 4. Risk aspects of resected polyps

Carcinoma, n		1
	≥10mm	1 (100%)
HGIN, n		7
	≤ 3mm	1 (14.3%)
	4-6mm	3 (42.9%)
	7-9mm	2 (28.6%)
	≥10mm	1 (14.3%)
SSA, n		8
	≤ 3mm	1 (12.5%)
	4-6mm	3 (37.5%)
	7-9mm	3 (37.5%)
	≥10mm	1 (12.5%)
LGIN, n		57
	≤ 3mm	11 (19.3%)
	4-6mm	20 (35.1%)
	7-9mm	14 (24.6%)
	≥10mm	12 (21.1%)
All resected polyps		125
	LGIN	105
	HGIN	7
	SSA	12
	Carcinoma	1
Patients ≥ 2 polyps		32
